# Prevalence of metallo-β-lactamase acquired genes among carbapenems susceptible and resistant Gram-negative clinical isolates using multiplex PCR, Khartoum hospitals, Khartoum Sudan

**DOI:** 10.1186/s12879-018-3581-z

**Published:** 2018-12-17

**Authors:** Mudathir Abdallah Adam, Wafa I. Elhag

**Affiliations:** Medical Microbiology Department, Faculty of Medical Laboratory Science, Al Neelain University, Khartoum, Sudan

**Keywords:** Metallo β-lactamase, *VIM*, *IMP*, *NDM*, Carbapenem, PCR, Gram-negative bacteria, Khartoum

## Abstract

**Background:**

The increased prevalence of carbapenem-resistant Gram-negative isolates caused by Metallo-β-lactamase (MBL) is worrisome in clinical settings worldwide. The mortality rate associated with infections caused by MBLs producing organisms ranging from 18 to 67%.

This study aimed to determine the prevalence of Metallo-β-lactamase genes among some Gram-negative clinical isolates (Carbapenems susceptible and resistant).

**Methods:**

This paper describes a descriptive cross-sectional study carried out to detect MBL genes such as (*blaVIM*, *blaIMP* and *blaNDM*) by multiplex PCR mixture reaction among 200 Gram-negative clinical isolates (*Citrobacter spp, Escherichia coli, Enterobacter spp, Klebsiella pneumoniae, Pseudomonas aeruginosa, Proteus mirabilis, Proteus valgaris*). Khartoum hospitals during 2015 to 2016.

Limitation: The study organisms were not evaluated for non-MBL carbapenemases, such as *KPC* and *OXA-48.*

**Results:**

The prevalence of MBL genes by multiplex PCR assays among 200 Gram-negative clinical isolates was 72(36.1%). MBL positive genes among 100 carbapenems sensitive and 100 resistant isolates were 27(27%) and 45(45%) respectively. There was a statistically, significant association between the antimicrobial susceptibility and the presences of MBL genes (*P.value* = 0.008).

*E.coli* was the predominant species possessing MBL genes 26(36.1%), with 22(30.7%) species having a combination of MBL genes.

*Verona integron Metallo beta-lactamase* (*VIM*) was the most frequent genes 28(38.9%) out of 72 MBL detected genes, followed by *imipenemase* (*IMP*) was 19(26.4%), and consequently, *New Delhi Metallo beta lactamase* was 3(4.2%).

**Conclusion:**

This study revealed a high prevalence of MBL genes in some Gram-negative isolates from Khartoum State Hospitals which were not previously established in these hospitals.

**Electronic supplementary material:**

The online version of this article (10.1186/s12879-018-3581-z) contains supplementary material, which is available to authorized users.

## Background

The production of hydrolytic β-lactamase enzymes is the most prevalent resistance mechanism towards β-lactam antibiotics [1, 2]. Metallo-β-lactamases constitute a worrisome group of enzymes, since they present a broad spectrum profile, hydrolyze penicillins, cephalosporins and carbapenems [1, 3], but not monobactams e.g.: aztreonam [4–7]. Carbapenem antibiotics are currently used as the last resort for treatment of the infections caused by multidrug-resistant Gram-negative bacteria [5, 7–9]. The mortality rate associated with MBL producers is reported to be from 18 to 67% [5, 6].

The most prevalent families of MBL resistant genes worldwide are *IMP* (inactivate imipenem, first reported in *Pseudomonas aeruginosa* strain from Japan in 1988) [10]. While the *VIM* (*Verona Integron-encoded Metallo-β-lactamase*) gene was first found in Europe, then emerged in other countries [7, 10]. However, *NDM*-producing bacteria were first isolated from a Swedish resident who contracted a urinary tract infection caused by carbapenem-resistant *K.pneumoniae* while he was in New Delhi in late 2007 [11–13]. Furthermore, the *NDM* gene later emerged in Pakistan, Indian subcontinent and the United Kingdom. Moreover, it represents a serious threat of rapid dissemination of multiple antibiotic resistance [4, 12, 14].

Furthermore, the easy and rapid dissemination of acquired MBL within a bacterial species is a major issue regarding the treatment of individual patients and confronting infection policies [15].

In a study done in Khartoum, Sudan late 2012 by Abdelrazig and his colleague among 74 clinical isolates of *Pseudomonas aeruginosa*, 57 isolates were recognized to have *IMP* family genes (*IMP-7* and *IMP-10*) using PCR assay [16].

Jamal W et al, (2013), reported in a study conducted in Mubarak Alkabeer hospital, Kuwait. There, out of 14 isolates of *Enterobacteriaceae* collected from intensive care units, eleven isolates produce *VIM-4*, and three isolates produced *NDM-1* [17].

The study reported here aimed to detect (*blaVIM*, *blaIMP* and *blaNDM*) Metallo-β-lactamase genes in Khartoum state.

## Methods

### Type and duration of the study

This was a descriptive cross-sectional study, carried out during January 2015–December 2016 at different hospitals, Khartoum State.

### Samples

A total of 200 samples of Gram-negative clinical isolates only susceptible and resistant to carbapenem antibiotics such as meropenem10μg and imipenem10μg were taken as part of the routine clinical procedure and stored for future analysis during six-month period from April 2015 to October 2015 from (Khartoum Teaching Hospital, Khartoum Pediatric Hospital, Omdurman Pediatric, El Blok Pediatric hospital, and Omdurman Medical Army Hospital) in Khartoum, Sudan. These strains were isolated from urine, wound swabs, sputum, blood, tissue aspirates, ear swab, and CSF samples.

Furthermore, the isolates were sub-cultured on cysteine electrolyte deficient (CLED) agar (HiMedia, India), and their identification was performed by biochemical tests such as oxidase test, Kligler iron agar (KIA), Indole test, citrate utilization, urease test and motility test.

### Antimicrobial susceptibility

Susceptibility to antibiotics was determined by Kirby-Bauer disk diffusion on Mueller Hinton agar (HiMedia, India). Ordinarily, the tested isolates were picked up with a sterile wire loop and suspended in peptone water (Mast Group, UK). In a good light, the turbidity of the suspension was prepared equivalent to 0.5 McFarland’s standard. A plate of Mueller Hinton agar was inoculated with the suspension using a sterile cotton swab. The swab evenly over the surface of the medium was streaked and meropenem (MEM) 10 μg and imipenem (IPM) 10 μg antimicrobial discs were placed. The plate was incubated aerobically at 35 °C for overnight [18, 19]. The inhibition zone diameters were measured by (mm) according to Clinical and Laboratory Standards Institute (CLSI) guidelines (2011) [19].

Thus, in this study carbapenem resistance was defined as testing non –susceptible to either meropenem and/ or imipenem. However, carbapenem susceptibility was defined as testing susceptible either to meropenem and/ or imipenem. Two strains were detected as intermediate and they considered resistant because the value of intermediate breakpoint was closer to the breakpoint of resistant.

### Multiplex PCR

MBL genes including (*blaVIM*, *blaIMP* and *blaNDM*) were screened among all isolates by multiplex polymerase chain reaction (PCR). Consequently, total DNA targeting both genomic and plasmid DNA was extracted with chloroform/ isopropanol chemical method. Specific primer for target DNA (MBL genes) designed by (Metabion/ Germany)**.**

*IMP*-F: (5^**ʼ**^GGA ATA GAG TGG CTT AAC TCT C 3^**ʼ**^) and IMP-R: (5^**ʼ**^ CGA ATG CGC ACC AG 3^**ʼ**^) 232 bp.

*VIM*-F: (5^**ʼ**^ TGG TGT TTG GTC GCA AT 3^**ʼ**^) and VIM-R: (5^**ʼ**^CGA ATG CGC ACC AG 3^**ʼ**^) 390 bp.

*NDM*-F: (5^**ʼ**^ CGG AAT GGC TCA TCA CGA TC 3^**ʼ**^) and NDM-R: (5^**ʼ**^ GGT TTG GCG ATC TGG TTT TC 3^**ʼ**^) 621 bp.

Every organism of the study population was tested with the three different primers in a single test.

Multiplex PCR amplifications were carried out in 20-μl volume containing 9-μl dH_2_O, 4 μl PCR Master Mix (Solis BioDyne, Estonia) ready to load, 3 μl of the three primer mixtures and 4-μl DNA template. The PCR amplifications were performed by thermo-cycler machine (TC-412, UK), using the following cycling parameters: one cycle (initial denaturation) 2 min at 95 °C, 30 cycles (denaturation) 30 Sec at 95 °C, 30 cycles (annealing) 30 Sec at 48 °C, 30 cycle (elongation) 30 Sec at 72 °C,and 2 min (final extension) at 72 °C. Moreover, the PCR product was resolved using 1.5% agarose gel electrophoresis to detect specific amplified product by comparing with standard molecular weight marker 50 base pairs (DNA ladder).

Limitation: The study organisms were not evaluated for non-MBL carbapenemases, such as *KPC* and *OXA-48.*

Data (Additional file 1) were analyzed by the Statistical package of social sciences (SPSS) programmed for Windows, version 16.

## Results

Different bacterial species were studied. Most were *E.coli* 71(35.5%) followed by *Pseudomonas aeruginosa* 54(27%), and less predominant organisms were *Citrobacter spp* and *Enterobacter spp* 1(0.5%) each, (Table 1).

**Table 1:** Distribution of clinical bacterial isolates (*n* = 200) according to their carbapenems susceptibility

Organisms	Sensitive No (%)	Resistant No (%)	Total No (%)
*E.coli*	45 (22.5)	26 (13)	71(35.5)
*P.aeruginosa*	21 (10.5)	33 (16.5)	54 (27)
*K.pneumoniae*	21 (10.5)	29 (14.5)	50 (25)
*P.mirabilis*	12 (6)	9 (4.5)	21 (10.5)
*P.valgaris*	–	2 (1)	2 (1.0)
*Enterobacter spp*	–	1 (0.5)	1 (0.5)
*Citrobacter spp*	1 (0.5)	–	1 (0.5)
Total No (%)	100 (50)	100(50)	200 (100)

The overall results revealed that 72(36.1%) from 200 clinical Gram-negative isolates, were positive for one or a combination of MBL genes. Consequently, MBL positive genes among 100 carbapenems sensitive and 100 resistant isolates were 27(27%) and 45(45%) respectively, the analysis showed statistically significant (*P.value* = 0.008) between carbapenem susceptibility and MBL genes (Table 2).

**Table 2:** Frequency rate of MBLs genes among Carbapenems sensitive and resistant Gram-negative isolates

Organisms	Positive MBLs genes No (%)	Negative MBLs genes No (%)	Total No (%)
Carbapenems resistant	45 (22.5)	55 (27.5)	100 (50)
Carbapenems sensitive	27 (13.5)	73 (36.5)	100 (50)
Total	72 (36)	128 (64)	200 (100)

*P.value* = 0.008

The MBL genes were heterogeneously distributed among the different species of Gram-negative isolates, with 22(30.7%) species having a combination of MBL genes. Moreover, *E.coli* was the predominant species possessing MBL genes 26(36.1%) (Table 3).

**Table 3:** Frequency rate of distribution of MBLs genes among the species of Gram-negative isolates

MBLs genes	Organisms					Total No (%)
	*K.pneumoniae* No (%)	*E.coli* No (%)	*P.aeruginosa* No (%)	*P.mirabilis* No (%)	*P.valgaris* No (%)	
VIM	7 (9.7)	12 (16.7)	8 (11.1)	1 (1.4)	0 (0)	28 (38.9)
IMP	8 (11.1)	6 (8.3)	4 (5.6)	1 (1.4)	0 (0)	19 (26.4)
NDM	0 (0)	2 (2.8)	1 (1.4)	0 (0)	0 (0)	3 (4.2)
VIM&IMP	0 (0)	2 (2.8)	1 (1.4)	0 (0)	0 (0)	3 (4.2)
VIM&NDM	3 (4.2)	2 (2.8)	6 (8.3)	0 (0)	0 (0)	11 (15.3)
IMP&NDM	1 (1.4)	1 (1.4)	2 (2.8)	0 (0)	0 (0)	4 (5.6)
VIM&IMP&NDM	1 (1.4)	1 (1.4)	1 (1.4)	0 (0)	1 (1.4)	4 (5.6)
Total	20 (27.8)	26 (36.1)	23 (31.9)	2 (2.8)	1 (1.4)	72 (100)

Key: VIM: Verona integron metallo-β-lactamase. IMP: Imipenemase. NDM: New Delhi metallo-β-lactamase

*Verona integron Metallo beta-lactamase* (*VIM*) was the most frequent gene detected in 28(38.9%) out of 72 positive MBLs genes, then, *imipenemase* (*IMP*) detected in 19(26.4%), and *NDM* reported in 3(1.5%) (Table 4).

**Table 4:** Frequency rate of MBL genes among the sensitive and resistant Gram-negative isolates

MBLs genes	Gram-negative isolates		Total No (%)
	Sensitive No (%)	Resistant No (%)	
VIM	12 (16.7)	16 (22.2)	28 (38.9)
IMP	12 (16.7)	7 (9.7)	19 (26.4)
NDM	0 (0)	3 (4.2)	3 (4.2)
VIM&IMP	1 (1.4)	2 (2.8)	3 (4.2)
VIM&NDM	1 (1.4)	10 (13.9)	11 (15.3)
IMP&NDM	1 (1.4)	3 (4.2)	4 (5.6)
VIM&IMP&NDM	0 (0)	4 (5.6)	4 (5.6)
Total	27 (37.5)	45 (62.5)	72 (100)

**Fig. 1:**
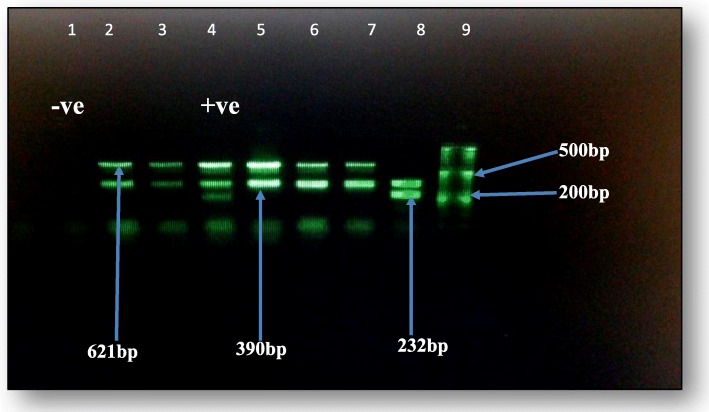
PCR product on Gel electrophoresis for MBLs genes among Gram-negative bacteria. Key. Lane 1: -ve control lanes 2, 3, 5, 6&7: 390 bp bla-_VIM_ & 621 bp bla-_NDM,_ and lane 4: (+ve control) 232 bp bla-_IMP,_ 390 bp bla-_VIM_ & 621 bp bla-_NDM._ Lane 8: 232 bp bla-_IMP_& 390 bp bla-_VIM_, Lane 9: DNA ladder 50 bp

Three possible Metallo β-lactamase (MBL) resistant genes (*blaVIM*, *blaIMP* and *blaNDM*), which detected in this study using PCR were illustrated on an agarose gel according to their amplicon size (Fig. 1).

## Discussion

Metallo-β-lactamase has emerged as a powerful resistance determinant in Gram-negative bacteria [20, 21], causing health problems of global dimensions [22–25] which can threatens human being with their newly built genetic structure [21], and play a principal role in drug resistance [22, 26, 27].

In general data on the dissemination of antimicrobial genes on sub-Saharan Africa is scarce, especially regarding the prevalence of MBL genes [28]. To our knowledge, this is not the first report of the presence of MBL encoding genes in Khartoum state, Sudan, a number of studies have been carried out, including a detection of *IPM* types in *Pseudomonas aeruginosa* performed by Abdelrazig and her colleague [16], *NDM* mediated carbapenem resistance was recently described by Mohamed et al. [29]. In addition to (*TEM*, *VIM*, *IMP*, *SHV*, *CTX,* and *KPC*) antimicrobial resistance genes among Gram-negative isolates were determined by Satir et al. [30]. By contrast, there are no published data regarding (*IPM*, *VIM* and *NDM*) carrying bacteria have been described in other regions of Sudan.

We believe this study represents the first report of the prevalence of (*IPM*, *VIM* and *NDM*) MBL encoding genes among Gram-negative isolates in Khartoum State, Sudan.

Furthermore, reports of emerging (*IPM*, *VIM* and *NDM*) MBL genes among Gram-negative strains have been published worldwide, including sub-Saharan Africa and Middle East countries such as Ethiopia [28], Kenya [11], Uganda [31], Egypt [32], South Africa [33], Saudi Arabia [2], Iraqi [4], Kuwait [17], and Lebanon [34].

MBL genes prevalence among carbapenem-resistant isolates was found 45(45%) out of 100, which agrees with MBL genes prevalence from Tanzania, Romania, India and Egypt by Mushi [5], Mereuţă et al, [35], Amudhan et al, [36], and Nelly, et al.*,* [37], they reported 42%, 46(43.4%), 92(51.4%) and 12(52.2%) respectively. This is a worrying result, probably due to the unjudged antibiotics use patterns in these countries.

However, our results were higher than studies conducted in Malaysia, Egypt, Uganda and Iraq by Khosravil [38] Zafer et al, [31], Okoche [39], and Anoar et al [4], they found 36, 31.3, 28.6%, and 39(22%) the prevalence of MBL genes respectively.

MBL genes prevalence among carbapenem sensitive isolates were found 27(27%) out of 100, this finding was higher than that found by Anoar et al, in Iraq, 7(3.9%) [4], ordinarily, MBL genes presence among carbapenems sensitive strains indicate that there might be a hidden MBL genes not detected by phenotypic tests, leading to the silent spread of these genes in the hospitals and the community.

This study revealed that MBL genes prevalence among carbapenem-resistant and sensitive isolates was (36.1%), which was agreed to that reported in Iraq, and Iran by, Anoar et al*,* [4], Aghamiri et al, [40] were 46(25,9%), and 90(42%), respectively. The difference may be due to the variation of geographical circulating strains.

*Verona integron Metallo-beta-lactamase* (*VIM*) was the most frequent gene represent 28 (38.9%) among 72 positive MBL genes, imipenemase (IMP) represent 19 (26.4%), and the NDM was the least detected in 3(1.5%).

Our results agree with studies done in Uganda, Tanzania, Egypt and Iran by Okoche et al*,* [39] Mushi [5], Zaferet al, [31] and Aghamiri [40], they reported the frequencies of (*VIM*, *IMP* and *NDM*) as: [21(10.7%), 12(6.1%) and 5(2.6%)], [34(15%), 28(12%), and 9(4%)], [58.3, 4.2 and 2.1%] and [70(33%) and 20(9%)] respectively. However, our findings were disagrees with that found in Iraq by Anoar et al, ^(4)^ they reported *IMP* was the most frequently detected gene 33(18.6%), *VIM* 19(10.7%), and *NDM* 2(1.12%) isolates.

*E.coli* was the most isolate harbours MBL genes 26(36.1%), followed by *Pseudomonas aeruginosa* 23(31.9%). Moreover, 22 isolates have harboured a combination of MBL genes. These findings disagree with Okoche *et al*, study, where *K.pneumoniae* was the most frequent MBL genes harbour 35(52.2%), and then 19(28.4%) *E.coli*, and 8 isolates of MBL genes positive harboured a combination of MBL genes similar to our findings [39].

## Conclusions

This study revealed a high prevalence of MBL genes in some Gram-negative isolates from Khartoum State Hospitals, all the three genes assayed (*blaVIM, blaIMP* and *blaNDM*) were detected in the study samples.

Detection of MBL genes among isolates which reported as sensitive to carbapenem was acting as a reservoir of such resistance genes with potential risk for the silent spread of these genes in hospitals and community.

This is the first report of the prevalence of (*VIM*, *IMP* and *NDM*) MBL genes in Gram-negative strains in Khartoum State, Sudan.

We recommend a regular screening and monitoring system should be set up to prevent the dissemination of these genes in the country.

## Additional file

Additional file 1: Samples, strains, PCR result information. (XLSX 18 kb)

## Abbreviations

bla: Beta-lactamase
CLED: Cysteine electrolytes deficient medium
CLSI: Clinical laboratory standard institute
*CTX*: Cefotaximase
DNA: Deoxyribonucleic acid
*IMP*: Imipenemase
*KPC*: Klebsiella pneumonia carbapenemases
MBL: Metallo-β-lactamase
*NDM*: New Delhi Metallo- β-lactamase
*OXA-48*: Oxacillinase-48
PCR: Polymerase chain reaction
*SHV*: Sulfhydryl variable
TC: Thermal cycler
*TEM*: Temoneira
UK: United Kingdom
*VIM*: Verona Metallo-β-lactamase
